# Synthesis of the biologically important dideuterium-labelled adenosine triphosphate analogue ApppI(*d*_2_)

**DOI:** 10.3762/bjoc.18.153

**Published:** 2022-10-14

**Authors:** Petri A Turhanen

**Affiliations:** 1 University of Eastern Finland, School of Pharmacy, Biocenter Kuopio, P.O. Box 1627, FIN-70211, Kuopio, Finland

**Keywords:** ApppI, ATP, deuterium labelling, HPCCC, mevalonate pathway, NMR, synthesis

## Abstract

The chemical synthesis of the dideuterium-labelled ATP analogue 1-adenosin-5’-yl-3-(3-methylbut-3-en-1,1-*d*_2_-1-ol) triphosphoric acid diester (ApppI(*d*_2_)) is described. ApppI has been reported to be an important mevalonate pathway metabolite, induced by nitrogen-containing bisphosphonates used for the treatment of several diseases related to the calcium metabolism, of which osteoporosis is the most well-known. The availability of ApppI(*d*_2_) opens possibilities to quantitative measurements of ApppI in biological samples by mass spectrometry. The synthesized target compound ApppI(*d*_2_) was purified by high-performance counter current chromatography and characterized by ^1^H, ^13^C, and ^31^P NMR spectroscopy as well as high-resolution mass spectrometry.

## Introduction

**Figure 1:**
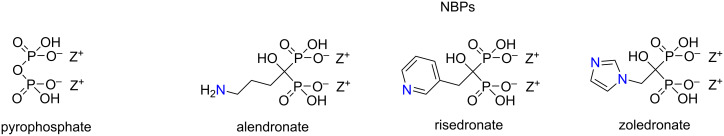
Structure of natural pyrophosphate and examples of NBPs (Z = Na or H).

**Figure 2:**
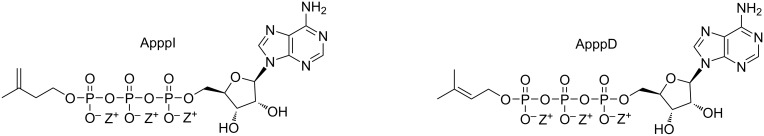
Structures of ATP analogues ApppI and ApppD (Z = Na or H).

It has become clear and evident that phosphonate chemistry plays a crucial role in drug research and development [1–4]. There are several phosphonate-containing compounds under research or already in clinical use as antiviral or anticancer drugs [2,5–6]. Bisphosphonates (BPs), the stable analogues of the natural pyrophosphate (Figure 1) found in cells, have been used for decades in the treatment of bone-related diseases, such as osteoporosis [7–8]. BPs can be categorized by the chemical structure into first-generation non-nitrogen-containing BPs (non-NBPs) and into second- and third-generation nitrogen-containing BPs (NBPs). Some examples of NBPs are alendronate, risedronate, and zoledronate (Figure 1) [9]. NBPs and non-NBPs have different mechanisms of action, and NBPs have been shown to prevent protein isoprenylation by inhibiting farnesyl pyrophosphate synthase in the mevalonate pathway. This process leads to the formation of a series of compounds of which the structures have been reported elsewhere [10–13]. In 2006, Mönkkönen et al. have reported that the use of NBPs led to the formation of ApppI, i.e., the isopentenyl ester of ATP (Figure 2), which may also isomerize to ApppD (Figure 2). The authors have also concluded that these compounds can act in two different ways: inhibition of the mevalonate pathway and blockade of mitochondrial ADP/ATP translocase, which is known to be involved in the induction of cell death [14]. The mechanism of action related to the antiresorptive and anticancer effects of NBPs has been proposed to be attributable to the metabolites formed in the mevalonate pathway induced by NBPs [15].

In 2020 in Finland, the wholesaling of BPs has been reported to amount to around 3.2 million EUR, and most were NBP sales. As such, it can be imagined that the worldwide sale is significant [16]. In light of the remarkable use of NBPs all over the world, and with the biological effects of ApppI already discovered, it is difficult to conceive how little is still known about this class of compounds: a recent search of the structure of ApppI on SciFinder led to only 18 references. It is not a whole truth, but it can be said that this is a very underexplored area of research. Interestingly, it has been reported that BP-based ATP analogs inhibit cell signaling pathways and that ApppI has a role in pain relief [17–18]. It can be proposed that some NBPs might represent a significant lead for the treatment of problematic chronic pain. Perhaps the main reason for the lack of research results on ApppI and ApppD is that these molecules are not commercially available, and in fact only few papers describe corresponding synthetic methods [19–22]. It is well known that labelled compounds (such as those with ^2^H and ^13^C labels) can be used as internal standards for the quantitative measurements of the equal compound without label, e.g., straight from the biological samples by MS [23–25]. This paper describes the synthesis of dideuterium-labelled ApppI(*d*_2_) in detail, which hopefully opens up possibilities for the develop of a quantitative MS method for the measurement of ApppI in (biological) samples, providing the research community with information related to biological functions of ApppI.

## Results and Discussion

**Scheme 1:**
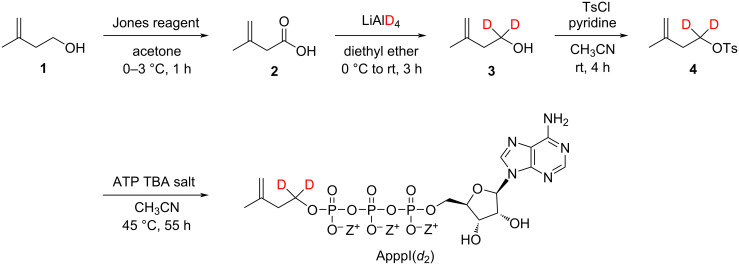
Synthetic route to target compound ApppI(*d*_2_) (Z = TBA).

The synthesis of the target compound ApppI(*d*_2_) started with commercially available 3-methylbut-3-en-1-ol (**1**), which was first oxidized to 3-methylbut-3-enoic acid (**2**) using freshly prepared Jones reagent as oxidizing agent. 3-Methylbut-3-enoic acid (**2**) was then reduced to 3-methylbut-3-en-1,1-*d*_2_-1-ol (**3**) with lithium aluminium deuteride, followed by tosylation with tosyl chloride. Tosylated 3-methylbut-3-en-1,1-*d*_2_-1-ol **4** was then treated with ATP TBA salt in acetonitrile at 45 °C for 55 h to give the target product ApppI(*d*_2_) (Scheme 1).

The first synthetic step from **1** to **2** was rather straightforward, leading to 50% yield of 3-methylbut-3-enoic acid (**2**) in about 88% purity, which was reasonable enough to continue with the second step. The synthesis of 3-methylbut-3-en-1,1-*d*_2_-1-ol (**3**) from **2** proceeded smoothly as expected. However, when evaporating the diethyl ether solvent from the final mixture, the concomitant evaporation of the desired product **3** was also noted, leading to only 31% yield. This is to be taken into account carefully when preparing 3-methylbut-3-en-1,1-*d*_2_-1-ol (**3**). In the final step to produce **4** from **3**, the main problem was the isolation. It was challenging to determine an appropriate eluent for TLC purification. Ultimately, using ethyl acetate/hexane 5:95 still led to only 24% yield. It can be said that the step from 3-methylbut-3-en-1-ol (**1**) to tosylated 3-methylbut-3-en-1,1-*d*_2_-1-ol **4** was not very efficient. However, according to the literature, this is the first example reported.

The procedure followed for the final synthetic step to ApppI(*d*_2_) and the detailed purification method by high-performance counter current chromatography (HPCCC) have previously been described elsewhere [21]. NMR spectroscopic methods were used to confirm the formation of the intermediate products **2**–**4** and the deuterated target compound ApppI(*d*_2_). The formation of **2** could easily be confirmed by the presence of carbonyl signal at 178 ppm in the ^13^C NMR spectrum. Further, the reduction of **2** by LiAlD_4_ led to the desired compound **3**, which was finally confirmed after the tosylation step and purification of compound **4**. The signal at 68 ppm with clear carbon–deuterium coupling (^1^*J*_CD_ = 22.9 Hz) in the ^13^C NMR spectrum was unambiguous proof of the doubly deuterated product **4**. ApppI(*d*_2_) was isolated using two different amounts of TBA salt. This was due to the solvation effect, which had already been reported earlier for the HPCCC purifications of ApppI [21] and ApppD [22]. A typical HPCCC chromatogram for the purification of ApppI(*d*_2_) is available in Supporting Information File 1.

## Conclusion

In conclusion, the synthesis of doubly deuterium-labelled highly important ATP analogue ApppI(*d*_2_) has been described in detail. This leaves the possibility to develop a quantitative MS method for the determination of ApppI in biological samples by using ApppI(*d*_2_) as internal standard. Samples of ApppI(*d*_2_) are available from the author upon request.

## Experimental

^1^H, ^31^P, and ^13^C NMR spectra were recorded on a 600 MHz spectrometer operating at 600.2, 243.0, and 150.9 MHz, respectively. The residual solvent signals (D_2_O, δ 4.79 ppm and CDCl_3_, δ 7.26 ppm) were used as references for ^1^H NMR measurements [26]. For ApppI(*d*_2_), the calibration peak used in the ^13^C NMR spectrum for characterization is mentioned in the data reported for ApppI(*d*_2_) below. The *^n^J*_CP_ couplings were calculated from the carbon spectra, with the coupling constant given in parentheses in Hz. The HPCCC purification method has previously been reported elsewhere and was used for the purification of ApppI(*d*_2_) [21]. HRMS spectra were recorded on a qTOF mass spectrometer using electrospray ionization (ESI) in negative mode. The purity of the products was determined from the ^1^H and ^31^P NMR spectra to be ≥95%, unless stated otherwise.

**ApppI(*d*_2_)⋅3.25 and ⋅5.25 TBA salts:** ATP disodium salt (269 mg, 0.49 mmol) was converted to the corresponding TBA salt by rapid treatment (5 min of stirring) with Dowex H^+^ cation exchange resin and the addition of 4 equiv of 40% TBAOH in H_2_O (1260 µL). The mixture was evaporated to dryness in vacuum and kept several days in vacuum to obtain a dry product. This ATP TBA salt was dissolved in dry CH_3_CN (3 mL), and 3-methylbut-3-en-1-yl-1,1-*d*_2_ 4-methylbenzenesulfonate (**4**, 130 mg, 0.54 mmol) was added. The reaction mixture stirred at 45 °C for 55 h before being evaporated to dryness in vacuum. The residue was stirred in diethyl ether (5 mL) for 10 min, and diethyl ether was removed. This was repeated twice. The residue was then dried in vacuum and purified by HPCCC following the method described in Reference [21]. When the appropriate fractions from the HPCCC purification were evaporated, the pure product was obtained in two different portions (70 mg as the ⋅5.25 TBA salt and 24 mg as the ⋅3.25 TBA salt, calculated total yield 11.4%, which was comparable to earlier results [20], see also HPCCC chromatogram in Supporting Information File 1). The product was obtained as very hygroscopic colorless foamy solid. NMR data for ApppI(*d*_2_)⋅3.25 TBA salt: ^1^H NMR (D_2_O) δ 8.52 (s, 1H), 8.21 (s, 1H), 6.08 (d, *J* = 6.1, 1H), 4.76–4.73 (m, 1H), 4.69–4.67 (m, 1H), 4.67–4.65 (m, 1H), 4.54–4.52 (m, 1H), 4.36–4.33 (m, 1H), 4.26–4.21 (m, 1H), 4.20–4.15 (m, 1H), 3.17–3.09 (m, 26H, from TBA salt), 2.25–2.18 (m, 2H), 1.61 (s, 3H), 1.63–1.54 (m, 26H, from TBA salt), 1.35–1.25 (m, 26H, from TBA salt), 0.90 (t, *J* = 7.3, 39H, from TBA salt); ^13^C NMR (D_2_O) δ 156.5, 153.7, 150.1, 144.3, 140.9, 119.5, 112.3, 87.6, 85.1 (d, ^2^*J*_CP_ = 9.3), 75.4, 71.5, 66.2 (d, ^3^*J*_CP_ = 5.4), 65.1 (m, ^2^H-C-^2^H, hardly visible), 59.0 (virtual t, from TBA salt), 38.5 (d, ^3^*J*_CP_ = 7.5), 24.1 (from TBA salt), 22.6, 20.1 (from TBA salt), 13.8 (from TBA salt, used as calibration peak: value marked same as in the earlier characterization of ApppI [20]); ^31^P NMR (D_2_O): δ −11.2 d (^2^*J*_PP_ = 19.4), −11.7 d (^2^*J*_PP_ = 19.4), −23.4 t (^2^*J*_PP_ = 19.4); HRMS–ESI (qTOF, *m*/*z*): [M − H]^−^ calcd for C_15_H_21_^2^H_2_N_5_O_13_P_3_, 576.0631; found, 576.0630. All NMR data were comparable to those reported elsewhere for conventional ApppI [19,21].

**Preparation of Jones reagent:** CrO_3_ (25 g) was slowly and carefully added to conc H_2_SO_4_ (25 mL) with stirring. After complete addition, the formed mixture was further added in very small portions to ice-cold water (75 mL) with vigorous stirring to form the final Jones reagent.

**Synthesis of 3-methylbut-3-enoic acid (2)** [27]**:** 3-Methylbut-3-en-1-ol (**1**, 3.0 mL, 2.56 g, 0.030 mol) was dissolved in acetone (150 mL), and the reaction mixture was cooled to 0–3 °C. Then, Jones reagent (15.6 mL) was added, and the reaction mixture was stirred at 0–3 °C for 1 h. The reaction mixture was made basic by adding an appropriate volume of 4 M NaOH with stirring (pH value measured by pH paper), and acetone was removed by evaporation in vacuum. The remaining mixture was acidified by addition of 6 M HCl and extracted with diethyl ether (3 × 15 mL). The combined diethyl ether fractions were dried over MgSO_4_ and filtered. After evaporation of the solvent, the final product was distilled in vacuum. The product was obtained as colorless liquid (1.5 g, 50%), bp 50–53 °C/2.0 mbar. According to the ^1^H NMR spectrum, the product purity was about 88%, which was considered to be pure enough to be used in the next step. ^1^H NMR (CDCl_3_) δ 4.95 (br, 1H), 4.89 (br, 1H), 3.08 (s, 2H), 1.84 (s, 3H); ^13^C NMR (CDCl_3_) δ 177.7, 138.1, 115.5, 43.2, 22.5.

**Synthesis of 3-methylbut-3-en-1,1-*d*_2_-1-ol (3)** [28]**:** LiAlD_4_ (320 mg, 7.6 mmol, 0.5 equiv) was added to dry and cold (0–3 °C) diethyl ether (15 mL) in an ice–water bath, and 3-methylbut-3-enoic acid (**2**, 1.5 g, 15 mmol), dissolved in dry diethyl ether (5 mL), was added in small portions with vigorous stirring. After completed addition, the ice–water bath was removed, and the reaction mixture was stirred for 3 h at room temperature before it was cooled again to 0–3 °C. Then, solid Na_2_SO_4_·10 H_2_O (1.0 g) was added, and the reaction mixture was stirred for 0.5 h at 0–3 °C. All solids were filtered off, and diethyl ether was removed slowly using a rotary evaporator in vacuum. The crude product was obtained as colorless liquid (415 mg, 31%). Most of the product had apparently been concomitantly evaporated with diethyl ether, and the remaining crude product still contained diethyl ether and side products. However, most of the obtained material was the desired product 3-methylbut-3-en-1,1-*d*_2_-1-ol (**3**), which was used directly in the next step. ^1^H NMR (CDCl_3_) δ 4.87 (br, 1H), 4.78 (br, 1H), 2.28 (s, 2H), 1.76 (s, 3H).

**Synthesis of 3-methylbut-3-en-1-yl-1,1-*d*_2_ 4-methylbenzenesulfonate (4)** [28]**:** 3-Methylbut-3-en-1,1-*d*_2_-1-ol (**3**, 415 mg, 4.7 mmol) was dissolved in dry acetonitrile (5 mL), and distilled pyridine (375 µL, 368 mg, 4.7 mmol) and tosyl chloride (900 mg, 4.7 mmol) were added. Then, the reaction mixture was stirred for 4 h at room temperature before being evaporated to dryness in vacuum. The residue was dissolved in diethyl ether, filtered, and evaporated to dryness in vacuum. The crude product (635 mg) was purified by preparative TLC using EtOAc/hexane 5:95 as eluent. The pure product was obtained at *R*_f_ = 0.11 (139 mg). However, also the fraction at *R*_f_ = 0.21 (181 mg) was observed to contain the desired product with some impurities, according to the ^1^H NMR spectrum. The fraction at *R*_f_ = 0.21 was further purified twice by analytical TLC, and the desired product was obtained purely with *R*_f_ = 0.27 (140 mg). The total amount of purified 3-methylbut-3-en-1-yl-1,1-*d*_2_ 4-methylbenzenesulfonate (**4**) was 279 mg (24%). ^1^H NMR (CDCl_3_) 7.79 (d, *J =* 8.0, 2H), 7.34 (d, *J =* 8.0, 2H), 4.79 (br, 1H), 4.67 (br, 1H), 2.45 (s, 3H), 2.34 (s, 2H), 1.66 (s, 3H);^13^C NMR (CDCl_3_) δ 144.9, 140.2, 133.3, 130.0 (2C), 128.0 (2C), 113.2, 68.0 (m, ^2^H-C-^2^H, ^1^*J*_CD_ = 22.9, hardly visible), 36.7, 22.5, 21.8.

## Supporting Information

File 1 ^1^H, ^13^C, and ^31^P NMR spectra as well as HPCCC chromatogram of ApppI(*d*_2_) purification.
