# Supplementary material for: Synthesis of the biologically important dideuterium-labelled adenosine triphosphate analogue ApppI(d2)
Source: Beilstein J Org Chem. 2022 Oct 14;18:1466–70. doi: 10.3762/bjoc.18.153 (PMC9577381; doi:10.3762/bjoc.18.153)
Supplement: File 1 — 1H, 13C, and 31P NMR spectra as well as HPCCC chromatogram of ApppI(d2) purification. [file Beilstein_J_Org_Chem-18-1466-s001.pdf]

BEILSTEIN JOURNAL OF ORGANIC CHEMISTRY

Supporting Information

for

Synthesis of the biologically important dideuterium-labelled adenosine triphosphate analogue ApppI(d_2)

Petri A. Turhanen

Beilstein J. Org. Chem. **2022,** *18,* 1466–1470. doi:10.3762/bjoc.18.153

^{1}H, ^{13}C, and ^{31}P NMR spectra as well as HPCCC chromatogram of ApppI(d_2) purification

License and Terms: This is a supporting information file under the terms of the Creative Commons Attribution License (https://creativecommons.org/licenses/by/4.0). Please note that the reuse, redistribution and reproduction in particular requires that the author(s) and source are credited and that individual graphics may be subject to special legal provisions.
The license is subject to the *Beilstein Journal of Organic Chemistry* terms and conditions: (https://www.beilstein-journals.org/bjoc/terms)

Table of contents

Structure of ApppI(d_2) S1

^{1}H, ^{31}P, and ^{13}C NMR spectra of ApppI(d_2)·3.25 TBA salt S2

Typical HPCCC chromatogram for the purification of ApppI(d_2) S4

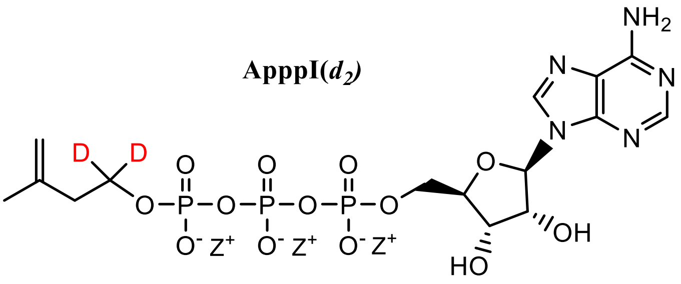

Structure of ApppI(d_2)

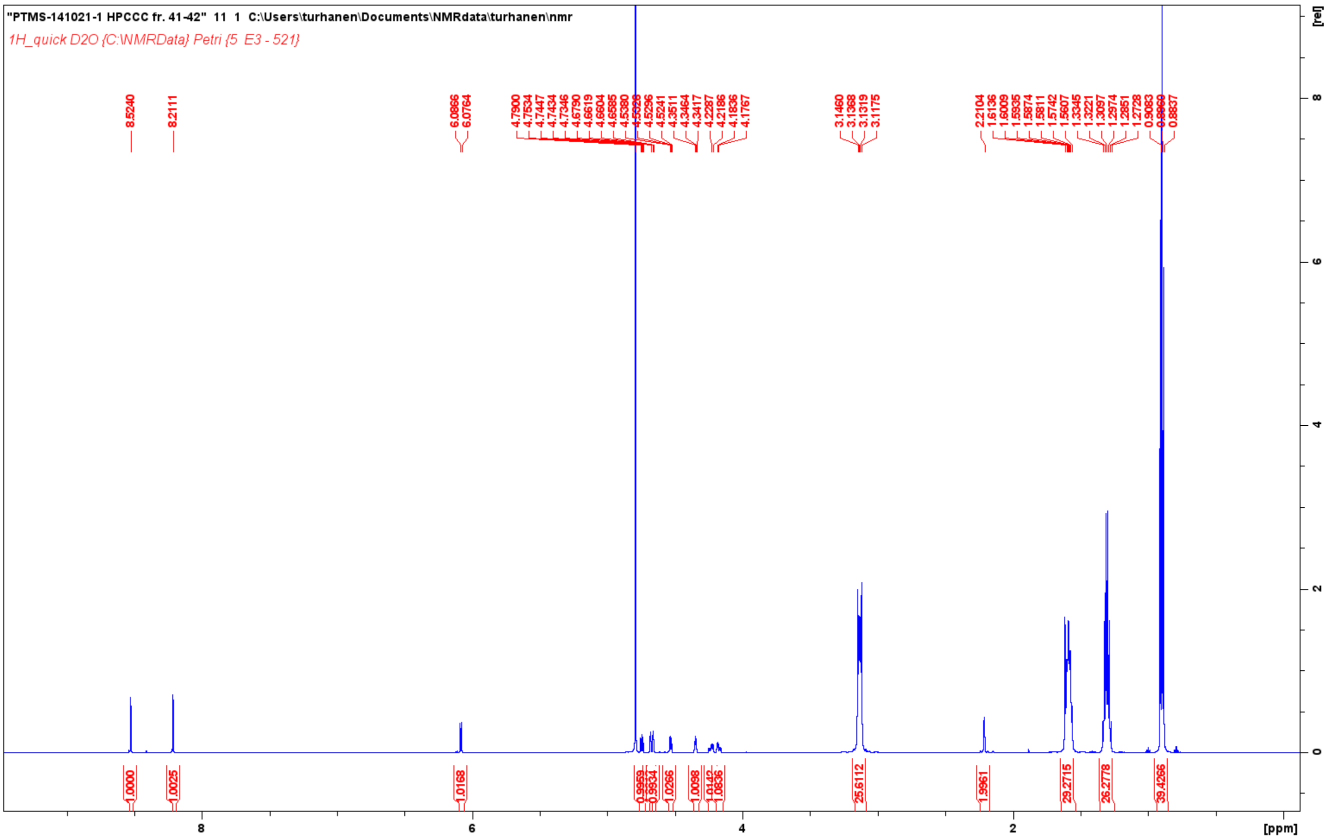

Figure S1: ^{1}H NMR spectrum of ApppI(d_2)·3.25 TBA salt.

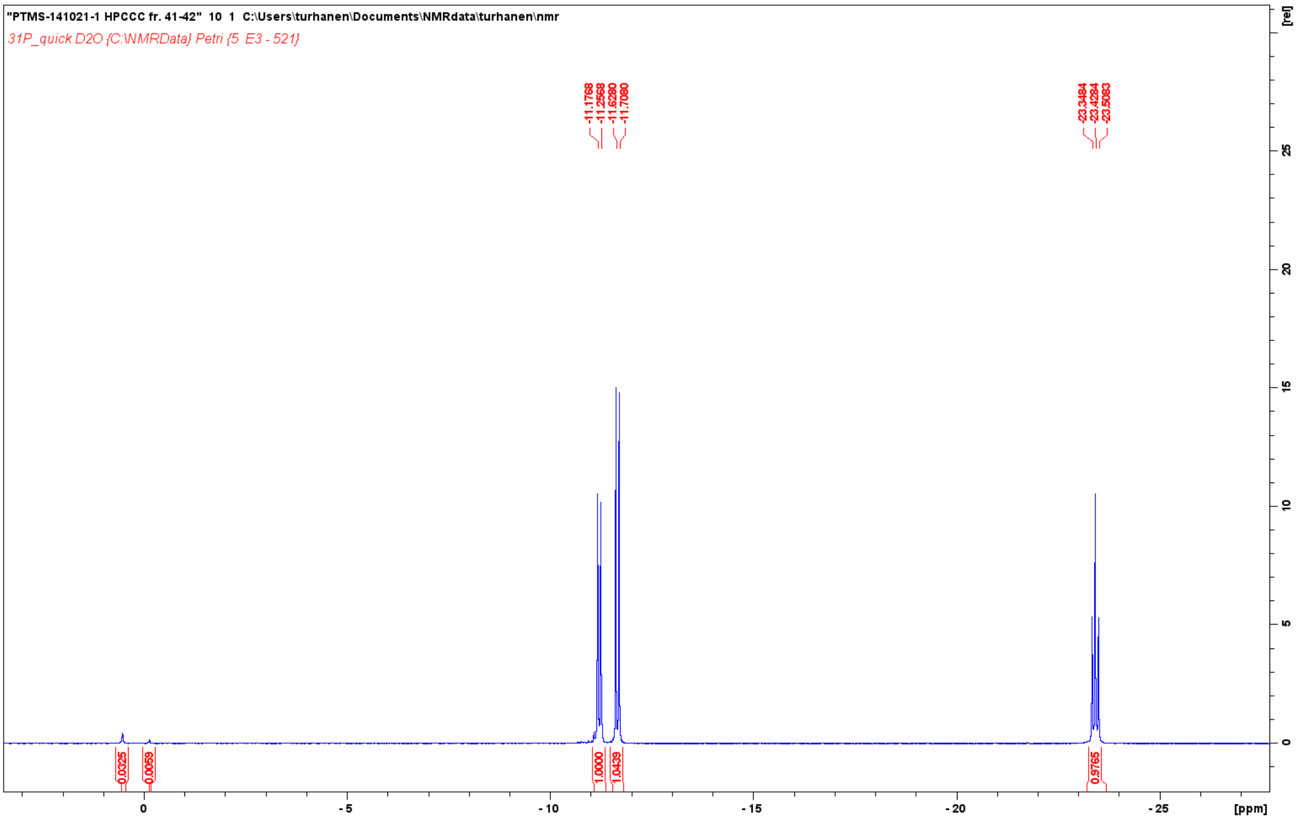

Figure S2: ^{31}P NMR spectrum of ApppI(d_2)·3.25 TBA salt.

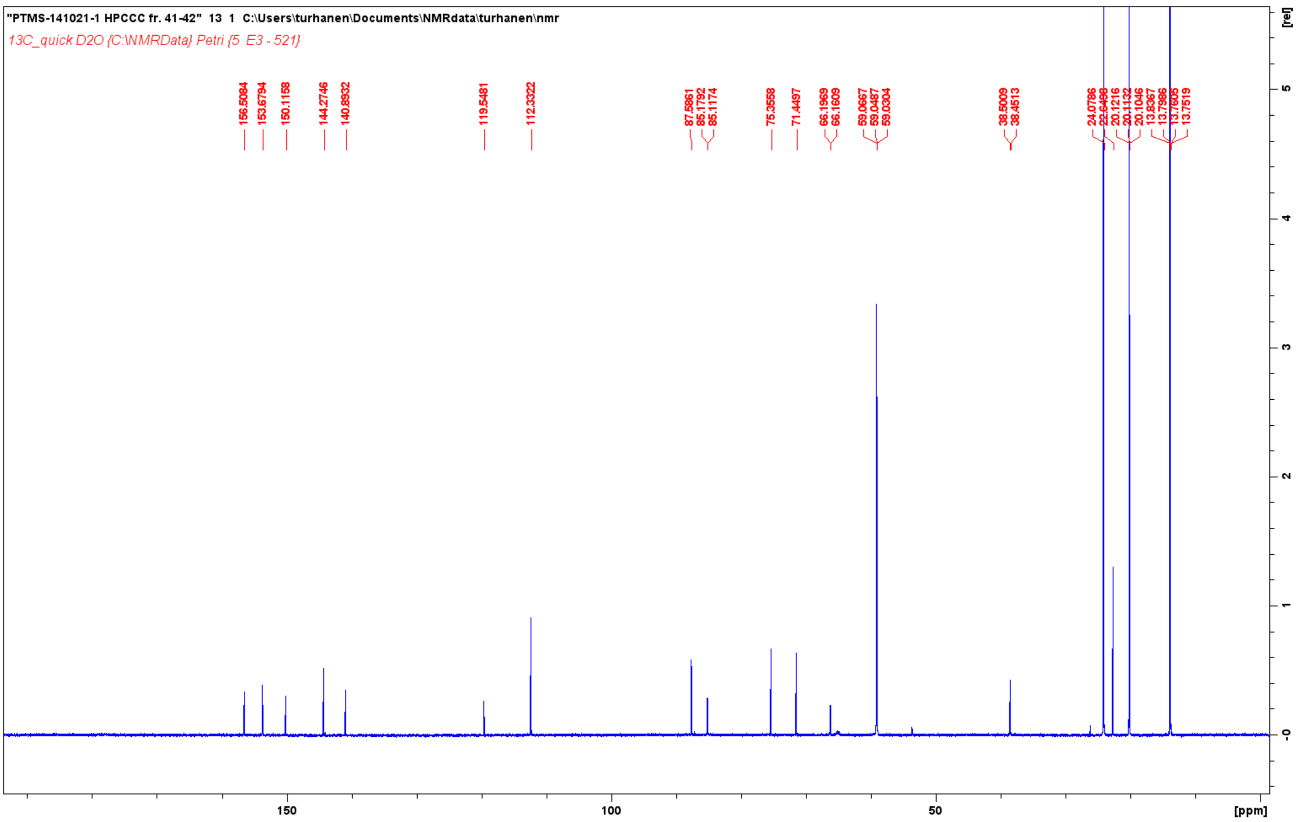

Figure S3: ^{13}C NMR spectrum of ApppI(d_2)·3.25 TBA salt.

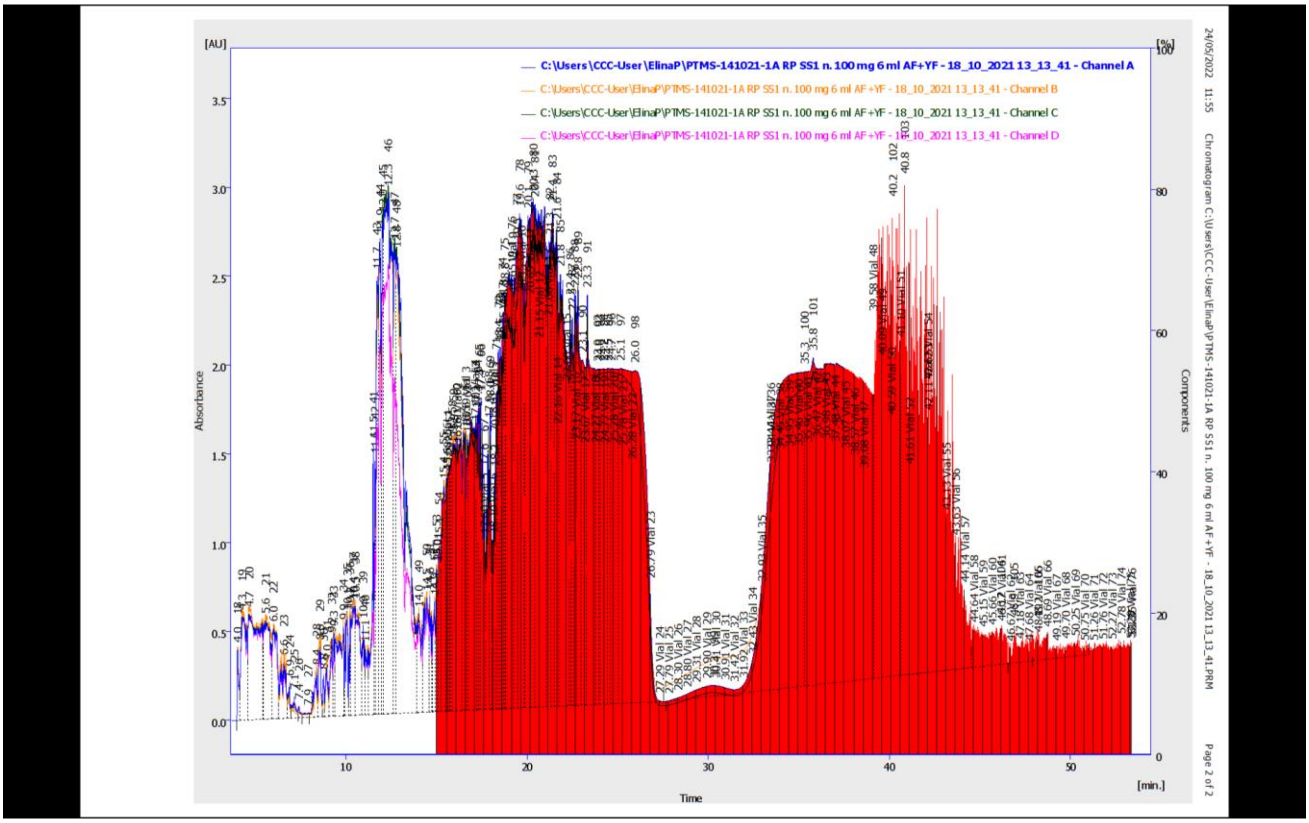

Figure S4: HPCCC chromatogram for the purification of ApppI(d_2). Vials 34–40 contained ApppI(d_2)·5.25 TBA salt and vials 41–42 ApppI(d_2)·3.25 TBA salt.
